# Uncommon case of mitochondrial disease: Mild amyotrophy of the legs and symmetrical lipomatosis of the arms

**DOI:** 10.1002/jimd.12820

**Published:** 2024-11-26

**Authors:** Leslie Bercu, Patrizia Amati‐Bonneau, Valérie Desquiret‐Dumas, Vincent Procaccio, François Maillot

**Affiliations:** ^1^ Department of Internal Medicine University Hospital of Tours, Univ Tours Tours France; ^2^ Department of Biochemistry and Molecular Biology CHU Angers Angers France; ^3^ Department of Genetics CHU Angers, Univ Angers, UMR CNRS 6015—INSERM U1083 Angers France; ^4^ UMR INSERM 1253 “IBraiN”, University of Tours Tours France

**Keywords:** lipomatosis, mitochondrial DNA mutations, mitochondial diseases

A 42‐year‐old woman was investigated for exercise intolerance. She already had a medical follow‐up because of a hypokinetic dilated cardiomyopathy identified at the age of 28 years and a chronic kidney disease, both secondary to a malignant hypertension episode. She complained about muscle pain and dyspnea on exertion, and was limited to a walking range of 200 m. She also experienced postexercise muscle pain. On clinical examination, no strength deficit was noted. She had mild amyotrophy of lower limbs and a symmetrical lipomatosis of her proximal upper limbs (Figure 1). No other localizations of lipomatosis were noted. Blood tests showed CK 1165 UI/L (26–192) and troponin 58.5 ng/L (3–14). The plasma redox cycle showed fasting lactate of 3 mmol/L, postprandial lactate up to 5.9 mmol/L along with a lactate/pyruvate ratio >20. Genetic tests revealed the presence of the pathogenic mitochondrial DNA (mtDNA) mutation m.8363A>G (MT‐TK)^1^ with a 45% mutant load in blood.

**FIGURE 1:**
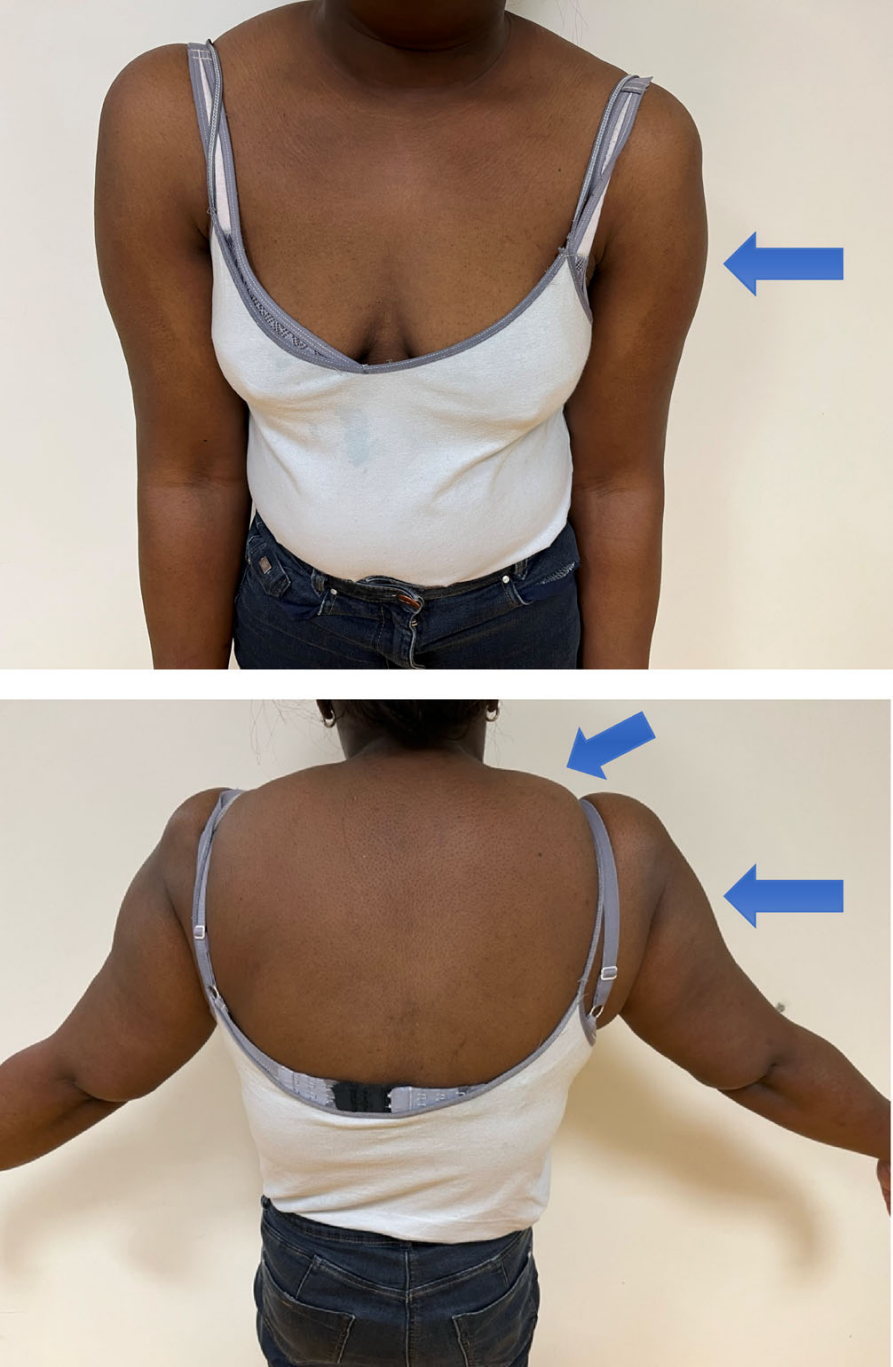
Lipomatosis of upper limbs in a patient with the mtDNA m.8363A>G mutation.

This case illustrates that lipomatosis can be a feature of mitochondrial disease, as shown by Musumeci et al., who reviewed 1300 patients reported in the database of the nationwide Italian Collaborative Network of Mitochondrial Disease.^2^ In this study, lipomatosis has been identified in 22 (1.7%) patients with primary mitochondrial disease. Interestingly, lipomatosis could be the sole manifestation of pathogenic mtDNA mutations. Among them, two patients carried the same mtDNAcm.8363 A>G variant as our patient, 18 harbored the m.A8344G>A variant and 2 patients had multiple deletions. Moreover, in a family study and a literature review, Virgilio et al. showed that mitochondrial DNA m.8363A>G mutation in the tRNALys gene was associated with a heterogeneous disease phenotype, including some rare cases of lipomas.^3^ As described in our clinical case, lipomatosis associated with mtDNA mutations is usually distributed in the upper body, affecting arms, shoulders, neck, and thoracic region.^1^ As multiple symmetric lipomatosis has a low prevalence of 1:25 000 in the general population and is a rare feature of mitochondrial disease, the case described here illustrates a very rare situation. Thus, it seems advisable to recommend mtDNA sequencing from blood and urine samples, mostly when the clinical picture is evocative of a mitochondrial disease, as in our observation. Regarding pathophysiology, Guallar et al. have shown that lipomatosis due to tRNA(Lys) mutations was associated with a pattern of altered expression of master regulators of adipogenesis consistent with enhanced proliferation of adipocytes, and with a distorted pattern of brown versus white adipocyte differentiation.^4^ In our case, such pattern could not be demonstrated because we did not perform any biopsy of the lipomas (considered as being nonrelevant for diagnosis).

To date, there is no curative therapy for most patients affected by mitochondrial diseases. However, supportive care, appropriate counseling, and regular follow‐up are prone to maintain or improve the quality of life of the affected patients. As recommended, our patient has been asked to maintain physical activity.^5^ After 2 years of follow‐up, her clinical state remained stable without any clinical change regarding lipomatosis.

## CONFLICT OF INTEREST STATEMENT

The authors declare no conflicts of interest.

## ETHICS STATEMENT

The present work has been conducted in accordance with to the ethical standards on human experimentation of our institution and with the Helsinki declaration of 1975, revised in 2013.
